# Lentiviral shRNA against KCa3.1 inhibits allergic response in allergic rhinitis and suppresses mast cell activity via PI3K/AKT signaling pathway

**DOI:** 10.1038/srep13127

**Published:** 2015-08-14

**Authors:** Hai Lin, Chunquan Zheng, Jing Li, Chen Yang, Li Hu

**Affiliations:** 1 Department of Otorhinolaryngology, Shanghai Jiao Tong University Affiliated Sixth People’s hospital, Shanghai, China; 2 Department of Otorhinolaryngology, Eye and ENT Hospital of Fudan University, Shanghai, China; 3 Department of Otorhinolaryngology, Hangzhou First People Hospital, Hanzhou, Zhejiang, China; 4 Department of Otorhinolaryngology, Rui-Jin Hospital of Shanghai Jiaotong University, Shanghai, China; 5 Central Laboratory, Eye and ENT Hospital of Fudan University, Shanghai, China

## Abstract

Calcium-activated potassium ion channel-3.1 (KCa3.1) plays a pivotal role in the potassium-calcium exchange involved in atopy. This study aimed to explore the impact of lentiviral-mediated shRNA silencing KCa3.1 on allergic response in a murine allergic rhinitis (AR) model. The BALB/c mice were divided into four groups: untreated AR group, negative control AR group, lentiviral KCa3.1-shRNA treated AR group and normal control group. Concentrations of ovalbumin (OVA)-specific IgE, histamine and leukotrienes C4 (LTC4) in serum, and IL-4, IL-9 and IL-17 in nasal lavage fluid (NLF) were analyzed. Goblet cells and mast cells were counted. KCa3.1 positive cells were counted after immunolabelling by immunofluorescence method. KCa3.1, Mucin 5AC (MUC5AC), and tryptase mRNA levels were determined using real-time polymerase chain reaction. Furthermore, P815 cell line was used to explore the role and mechanism of lentiviral KCa3.1-shRNA on mast cells. The results showed that LV-KCa3.1-shRNA intervention effectively attenuated allergic responses in LV-KCa3.1-shRNA treated mice. LV-KCa3.1-shRNA intervention effectively suppressed KCa3.1 levels and phosphorylation of AKT in P815 cells, leading to the downregulation of tryptase, IL-6 and IL-8 levels. LV-KCa3.1-shRNA intervention effectively attenuated the allergic responses in AR and suppressed mast cell activity by inhibiting PI3K/AKT signaling pathway.

Allergic rhinitis (AR) is a chronic inflammatory upper airway disease characterized by sneezing, rhinorrhea and stuffy nose, and AR could negatively impact patient’s quality of life^1^,^2^. There is ample evidence that goblet cell hyperplasia, mast cell infiltration and lymphocytes disorders are involved in the pathogenesis of AR^3^,^4^,^5^. However, the precise pathogenesis of AR remains uncertain. Accumulating evidences indicate that antihistamine agents and corticosteroids are effective in the treatment of AR^6^,^7^, however, the transient efficacy and drug tolerance of them make AR not easy to cure^8^. It is acknowledged that allergen immunotherapy (also named as desensitization) can alleviate allergic symptoms by inducing specific immunologic tolerance in the AR sufferer with one specific allergen, however, many patients are allergic to more than one allergen, and this treatment is time-consuming and expensive, and it has the risk of triggering serious allergic reactions. Hence, a novel safe, effective and inexpensive treatment for AR is urgently needed.

RNA interference (RNAi) is a gene-specific RNA degradation process mediated by short interfering RNA (siRNA) and is a promising therapeutic tool directed against a potential therapeutic target in the allergic disorders, infectious diseases and cancers^9^,^10^,^11^. A short hairpin RNA (shRNA) is a precursor of siRNA and is composed of siRNA sequence and hairpin loop sequence^12^,^13^. It has been established that shRNA was more stable than siRNA and was more readily integrated into the plasmid or viral vectors^14^. Lentivirus-mediated shRNA using lentiviral vector yielded an efficient and stable response of the target gene silencing both *in vitro* and *in vivo*^15^.

It is well established that calcium ion (Ca^2+^) is essential for regulating most cell activities involving cell growth, transformation, and migration. There is ample evidence that upregulation of intracellular Ca^2+^ participates in the proliferation of airway epithelial cells, smooth muscle cells and lymphocytes involved in asthma^16^,^17^,^18^. It has been illustrated that sustained Ca^2+^ influx through Ca^2+^ release-activated Ca^2+^ channel (CRAC) requires sufficient electrical driving force which is conserved by efflux of potassium ion (K^+^) and intermediate Ca^2+^-activated K^+^ channel (KCa3.1, also named as KCNN4) has been proven to mainly modulate the efflux of K^+^ and plays a pivotal role in the potassium-calcium exchange involved in the allergic and inflammatory responses^19^. Nevertheless, whether KCa3.1 is involved in the pathophysiology of AR and the inhibition of KCa3.1 could suppress the allergic response in AR are unclear. Hence, the present study was aimed to explore the expression and role of KCa3.1 in the pathogenesis of AR and to further assess the therapeutic effect of lentivirus-mediated shRNA silencing of KCa3.1 in a murine AR model. Furthermore, P815 cell line was used to explore the role and mechanism of lentiviral KCa3.1-shRNA on mast cells.

## Results

### Effects of LV-KCa3.1-shRNA on nasal symptoms in a murine AR model

**Figure 1:**
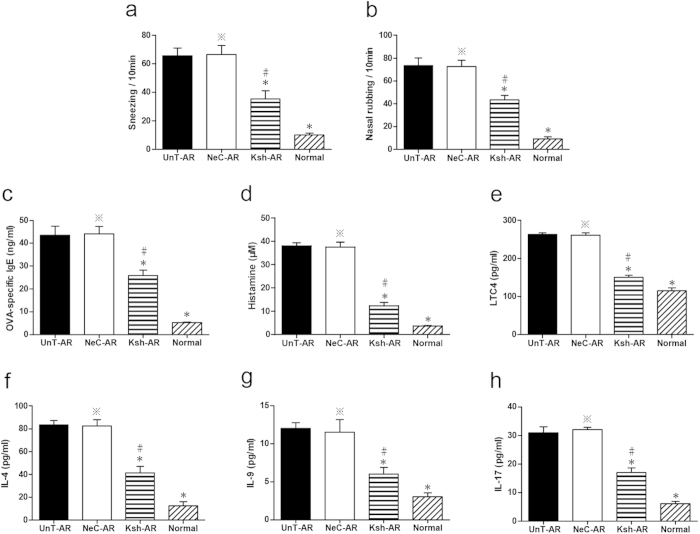
Sneezes and nasal rubbing in UnT-AR group, NeC-AR group, Ksh-AR and normal control group. (**a**–**b**). Concentrations of OVA-specific IgE, histamine and LTC4 in serum, and IL-4, IL-9 and IL-17 in NLF assayed by ELISA (**c**–**h**). One-way ANOVA test was employed for intergroup comparison. *P < 0.01 vs UnT-AR group. #P < 0.01 vs NeC-AR group. ^*^P > 0.05 vs UnT-AR group.

As indicated in Fig. 1a,b, significant elevation of frequencies of sneezes and nasal rubbing were found in untreated AR mice compared with normal control mice, respectively (P < 0.01), and LV-KCa3.1-shRNA group showed lower frequencies of sneezes and nasal rubbing compared with untreated AR or negative control group, respectively (P < 0.01).

### Effects of LV-KCa3.1-shRNA on OVA-specific IgE, histamine and LTC4 in serum and cytokine levels in NLF

To further illustrate the roles of LV-KCa3.1-shRNA on mast cell, Th2, Th9 and Th17 cells in AR, serum OVA-specific IgE, histamine and LTC4, IL-4 (Th2-derived cytokine), IL-9 (Th9-derived cytokine) and IL-17 (Th17-derived cytokine) levels in NLF were assayed by ELISA. As illustrated in Fig. 1c–h, significantly elevation of serum OVA-specific IgE, histamine and LTC4 levels, and IL-4, IL-9 and IL-17 levels in NLF from untreated AR group were found compared with normal control mice. LV-KCa3.1-shRNA treatment resulted in the reduction of OVA-specific IgE, histamine, LTC4, IL-4, IL-9 and IL-17 levels in LV-KCa3.1-shRNA treated group compared with untreated AR or negative control group, respectively (P < 0.01). These findings were consistent with the changes in the nasal symptoms following LV-KCa3.1-shRNA treatment.

### Goblet cell hyperplasia, mast cell infiltration and KCa3.1 immunoreactivity in nasal tissues

**Figure 2:**
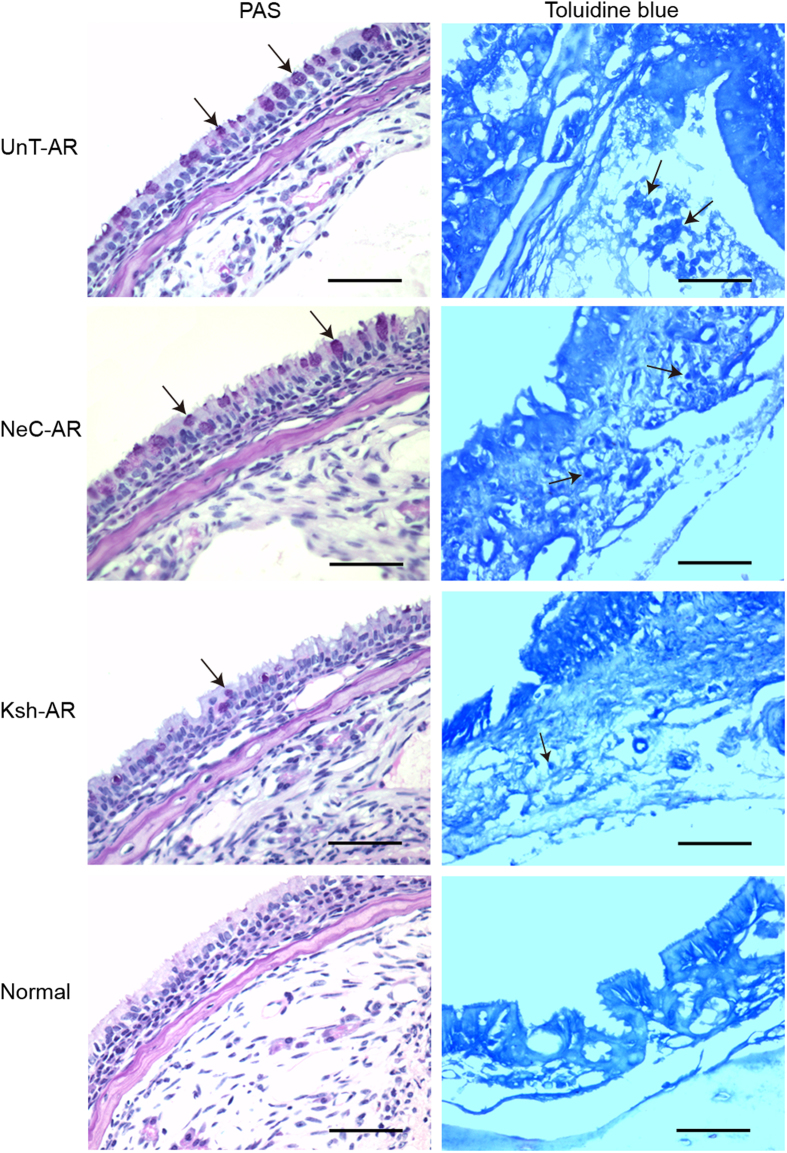
Goblet cell and mast cell numbers in UnT-AR group, NeC-AR group, Ksh-AR and normal control group. Arrows indicate goblet cell or mast cell. Scale bar = 50 μm.

**Figure 3:**
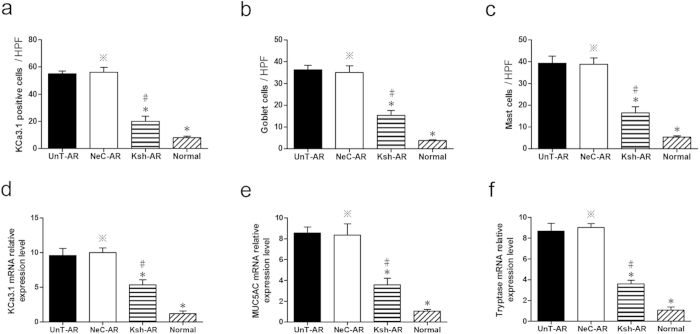
Analysis of KCa3.1 protein immunolabelling, goblet cell and mast cell numbers. (**a**–**c**) KCa3.1, MUC5AC and tryptase mRNA levels assayed by real-time PCR (**d**–**f**). One-way ANOVA test was employed for intergroup comparison. *P < 0.01 vs UnT-AR group. #P < 0.01 vs NeC-AR group. ^*^P > 0.05 vs UnT-AR group.

As indicated in Fig. 2 and Fig. 3b,c, significant elevation of goblet cell and mast cell numbers were found in untreated AR mice compared with normal control mice (P < 0.01), and LV-KCa3.1-shRNA group showed less goblet cell and mast cell numbers compared with untreated AR or negative control group, respectively (P < 0.01), indicating that LV-KCa3.1-shRNA treatment reduced goblet cell hyperplasia and mast cell infiltration in AR mice.

**Figure 4:**
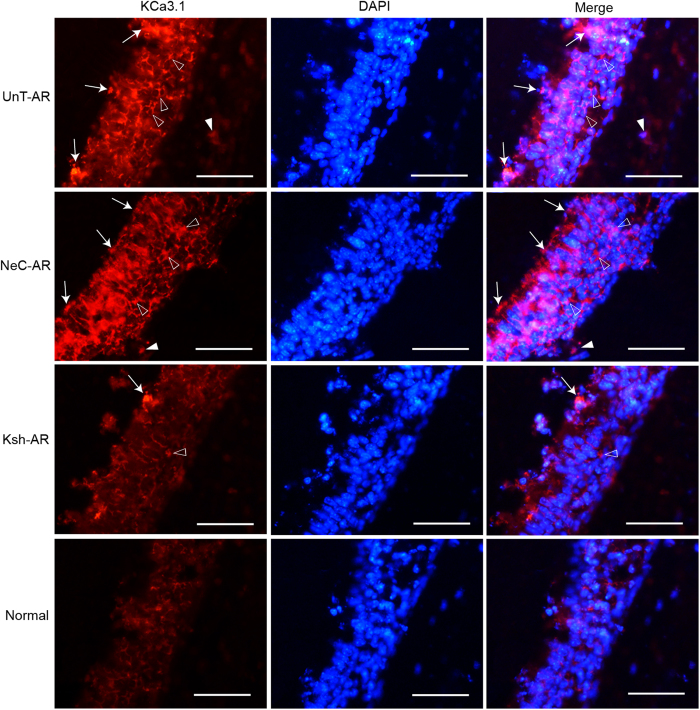
KCa3.1 immunolabelling assayed by immunofluorescence in UnT-AR group, NeC-AR group, Ksh-AR and normal control group. Arrows indicate KCa3.1 positive mucosal epithelial cells. Empty triangles indicate KCa3.1 positive epithelial cells surrounding basal lamina. Filled triangles indicate KCa3.1 positive sub-mucosal inflammatory cells. Scale bar = 50 μm.

As depicted by immunofluorescence staining (Fig. 4), KCa3.1 positive cells were mainly epithelial cells among mucosal surface and surrounding basal lamina, and sub-mucosal inflammatory cells in the nasal mucosa. KCa3.1 was significantly overexpressed in AR group compared with control group; notably, LV-KCa3.1-shRNA treatment resulted in the reduction of KCa3.1 protein levels in LV-KCa3.1-shRNA treated group compared with untreated AR or negative control group, respectively (P < 0.01). These results were consistent with the changes in the nasal symptoms and cytokine levels following LV-KCa3.1-shRNA treatment.

Moreover, KCa3.1 positive cell numbers were positively correlated with goblet cell or mast cell numbers respectively (Spearman’s test, r = 0.821, p = 0.023 and r = 0.865, p = 0.012).

### Effects of LV-KCa3.1-shRNA on KCa3.1, MUC5AC and tryptase mRNA levels in AR mice

To further illuminate the roles of LV-KCa3.1-shRNA on goblet cell and mast cell in AR mice, mRNA levels of KCa3.1, MUC5AC, which is mainly secreted by goblet cell^20^, and tryptase, known as a key mast cell marker which mainly exists in the secretory granules of mast cell^21^, were assayed by real-time PCR.

As showed in Fig. 3d–f, significant upregulation of KCa3.1, MUC5AC and tryptase mRNA levels were found in AR mice compared with normal control mice (P < 0.01); Of note, LV-KCa3.1-shRNA treatment suppressed KCa3.1, MUC5AC and tryptase mRNA levels in LV-KCa3.1-shRNA treated group compared with untreated AR or negative control group, respectively (P < 0.01). Moreover, KCa3.1 mRNA level was positively correlated with MUC5AC or tryptase mRNA level, respectively (Spearman’s test, r = 0.738, p = 0.037 and r = 0.833, p = 0.01). These results were consistent with the findings of histopathologic examination following LV-KCa3.1-shRNA treatment.

### Effects of LV-KCa3.1-shRNA on KCa3.1 levels and phosphorylation of AKT in P815 cells

**Figure 5:**
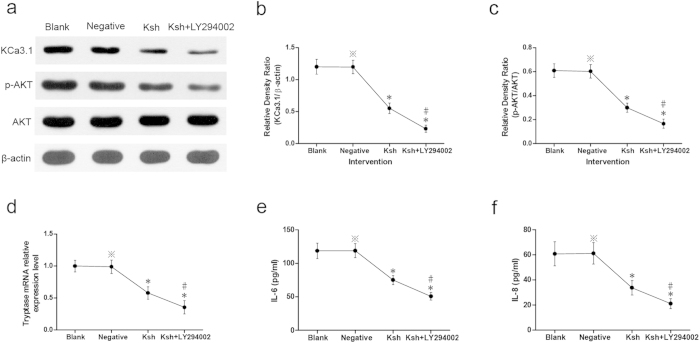
Results of P815 cell culture and intervention experiments. Western blotting analysis of KCa3.1 levels (KCa3.1/β-actin) and phosphorylation of AKT (p-AKT/AKT) (**a**–**c**) tryptase mRNA levels assayed by real-time PCR (**d**). Concentrations of IL-6 and IL-18 in the culture supernatants (**e**–**f**). One-way ANOVA test was employed for intergroup comparison. *P < 0.01 vs blank control group. #P < 0.01 vs Ksh group. ^*^P > 0.05 vs blank control group. The cropped blots images are shown in the figure and the full-length blots images are presented in Supplementary Information.

To further illustrate the roles and mechanism of LV-KCa3.1-shRNA on mast cell, P815 cell line was intervened with LV-KCa3.1-shRNA and LY294002 serving as a specific inhibitor of PI3K/AKT signaling pathway. As indicated in Fig 5a–c, strong bands of KCa3.1 and p-AKT protein were found in the blank control or negative control group, respectively; whereas weak bands were detected in LV-KCa3.1-shRNA group or LV-KCa3.1-shRNA +LY294002 group; notably, weaker bands were found in LV-KCa3.1-shRNA +LY294002 group compared with LV-KCa3.1-shRNA group. KCa3.1 levels (KCa3.1/β-actin) and phosphorylation of AKT (p-AKT/AKT) were suppressed by LV-KCa3.1-shRNA, and LY294002 enhanced these effects.

### Effect of LV-KCa3.1-shRNA on tryptase mRNA level in P815 cells

As showed in Fig. 5d, significant downregulation of tryptase mRNA level was found in LV-KCa3.1-shRNA group or LV-KCa3.1-shRNA+LY294002 group (P < 0.01), indicating that P815 cells were inactivated by LV-KCa3.1-shRNA and LY294002 enhanced the inhibition.

### Effects of LV-KCa3.1-shRNA on cytokines released from P815 cells

As showed in Fig. 5e,f, significant downregulation of IL-6 and IL-8 levels were found in LV-KCa3.1-shRNA group or LV-KCa3.1-shRNA+LY294002 group (P < 0.01), indicating that IL-6 and IL-8 levels released from P815 cells were suppressed by LV-KCa3.1-shRNA and LY294002 enhanced the inhibition.

## Discussion

It has been proved that goblet cell hyperplasia, mast cell infiltration, Th-2, Th-9 and Th-17 polarization are recognized as important features of AR^3^,^5^,^22^,^23^. In the present study, significantly increased goblet cell hyperplasia, mast cell infiltration, IL-4, IL-9 and IL-17 levels were found in AR mice compared with normal control mice, which is consistent with previous reports^3^,^5^,^22^,^23^. In addition, MUC5AC and tryptase mRNA levels were significantly upregulated in AR mice compared with normal control mice, further confirming that goblet cell hyperplasia and mast cell infiltration are involved in the pathogenesis of AR.

Our previous reports demonstrated that Orai1, an essential component of the CRAC was upregulated and led to elevation of intracellular Ca^2+^ concentration in a murine AR model and target inhibition of Orai1 could effectively alleviate murine AR through reducing Ca^2+^ influx^24^,^25^. Recently, there is emerging evidence that KCa3.1 has been implicated as an amplifier of CRAC current and could sustain sufficient membrane potential and electrochemical driving force for Ca^2+^ influx by modulating K^+^ efflux^26^,^27^. Previous studies have demonstrated that targeted inhibition of KCa3.1 gene could effectively suppress the allergic responses in asthma and inhibit cell proliferation in cancers^28^,^29^. In our present study, as indicated in Figs 1, 2, 3 and Fig. 3a–f, LV-KCa3.1-shRNA could effectively alleviate allergic symptoms of AR mice by reducing goblet cell hyperplasia, mast cell infiltration, OVA-specific IgE, histamine, LTC4, IL-4, IL-9, IL-17, KCa3.1, MUC5AC and tryptase levels. These results show that LV-KCa3.1-shRNA intervention may suppress goblet cell hyperplasia and mast cell infiltration, and inactivate plasma cells, Th2 ,Th9 and Th17 cells.

Considerable data indicate that mast cells play central roles in the pathologic change of nasal mucosal tissue and pathophysiologic process in AR^30^,^31^,^32^. Accumulating evidence has shown that upregulation of intracellular Ca^2+^ concentration is essential for mast cell activation^33^,^34^ and treatment of mast cells with carbon dioxide suppresses degranulation via repression of intracellular calcium levels^35^. KCa3.1 channels are required for mast cell recruitment and activation^36^. To date, there are many lines of evidence indicating that PI3K/AKT signaling pathway plays an essential role in the maturation and activation of mast cells^37^,^38^,^39^ and PI3K/AKT signaling pathway inhibitor LY294002 suppresses mast cells degranulation and activation^40^,^41^. In the present study, to further illustrate the roles and mechanism of LV-KCa3.1-shRNA on mast cell, P815 cell line was treated with LV-KCa3.1-shRNA and PI3K/AKT signaling pathway inhibitor LY294002. As depicted in Fig. 5a–f, LV-KCa3.1-shRNA group showed lower levels of KCa3.1, AKT phosphorylation, tryptase, IL-6 and IL-8 compared with blank control or negative control group, respectively and LY294002 enhanced these effects. These results indicate that the roles of LV-KCa3.1-shRNA on mast cells may be mediated via inactivation of the PI3K/AKT signaling pathway in mast cells. Our findings are in agreement with previous findings demonstrating that PI3K/AKT signaling pathway inhibitor LY-294002 exerted inhibitory effects on the mast cell activation^41^,^42^,^43^.

Some weaknesses and limitations of our study should be interpreted. First, relatively small sample size was included in this study and studies with large sample sizes are warranted to further clarify these findings. Second, it is necessary to perform whole genome microarray to assess the off target effects of LV-KCa3.1-shRNA in future studies.

In conclusion, LV-KCa3.1-shRNA could effectively alleviate murine allergic rhinitis by suppressing KCa3.1, leading to the reduction of goblet cell hyperplasia and mast cell infiltration, as well as attenuation of allergic responses and inflammatory factors. The role of LV-KCa3.1-shRNA on mast cells may be mediated via inactivation of the PI3K/AKT signaling pathway in mast cells. The targeted inhibition of KCa3.1 gene mediated by lentiviral vector may be a novel promising approach in AR gene therapy.

## Methods

### Construction of lentiviral KCa3.1-shRNA vector

**Figure 6:**
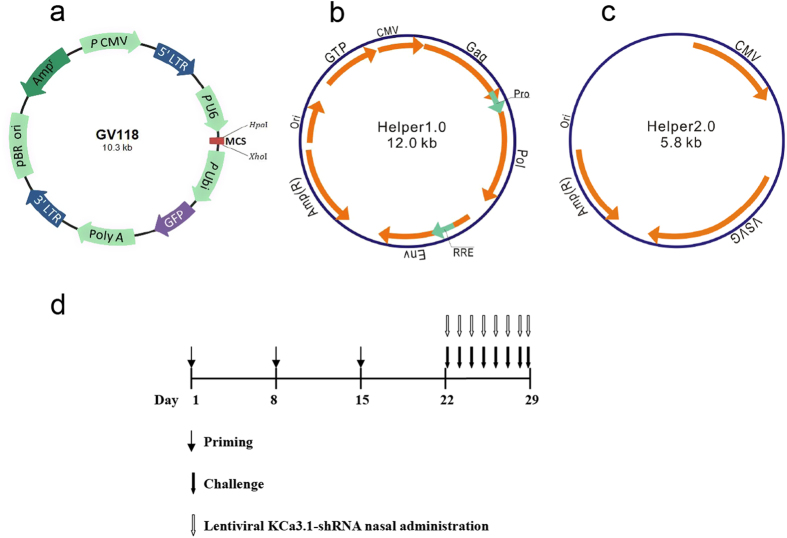
Lentiviral vector system. (**a**–**c**) GV-118 vector encoding sustained RNA expression (**a**) pHelper 1.0 vector, encoding a viral structural protein (**b**) and pHelper 2.0 vector, encoding a viral capsid protein (**c**). Schematic diagram of a murine AR model and LV-KCa3.1-shRNA treatment (**d**).

The shRNA targeting sequence for KCa3.1 (KCa3.1-shRNA) was 5′-TCATGATGGACATCCATTA-3′ and the scrambled RNA sequence (negative-shRNA) used as negative control was 5′-TTCTCCGAACGTGTCACGT-3′. The GV118 vector encoding KCa3.1-specific shRNA, pHelper 1.0 vector, and pHelper 2.0 vector (Fig. 6a–c, GeneChem, Shanghai, China) were co-transfected into the 293T cell line using lipofectamine 2000 (Invitrogen, Carlsbad, USA) to generate re-constructed lentiviruses according to the manufacturer’s instructions. After 48 hours, the supernatants were collected and re-constructed lentiviruses were harvested by centrifugation and then stored at −80 °C for further experiments.

### Murine AR model and LV-KCa3.1-shRNA treatment

A total of 48 female BALB/c mice (6–8 weeks of age, Shanghai Experimental Animal Center, Shanghai, China) were included in this study and they were kept in a specific-pathogen free facility. Animal experimental protocols were approved by the Animal Care and Use Committee of Eye and ENT Hospital of Fudan University and the experiments were carried out in accordance with the approved guidelines. The mice were divided four groups (n = 12 per group): untreated AR (UnT-AR) group, negative control AR group (NeC-AR), lentiviral KCa3.1-shRNA treated AR group (LV-KCa3.1-shRNA treated AR group, Ksh-AR) and normal control group. The murine AR model was established as described previously^24^. Briefly, mice were sensitized with 0.2 mL suspension comprising 0.5 mg/mL ovalbumin (OVA, Sigma-Aldrich, USA) and 20 mg/mL aluminum hydroxide (Sinopharm Chemical Reagent Co. Ltd., China) by intraperitoneal injection on Day 1, 8, and 15, respectively. Then, the mice were challenged daily with 20 μl OVA solution (40 mg/ml in normal saline) by intranasal instillation on Days 22 to 29 (Fig. 6d). To improve gene transfection efficiency, the mice were instilled intranasally with 4μl lysophosphatidylcholine (1% w/v in PBS, Sigma-Aldrich, USA) prior to delivery of the lentiviral vector system, as described previously^44^. Mice in lentiviral KCa3.1-shRNA treated group were instilled intranasally with lentivirus mediated KCa3.1-shRNA (20 μl, 1 × 10^8^ TU/ml) and the mice in negative control group were given with the same dose of lentivirus mediated negative-shRNA.Mice in normal control group were administrated with normal saline.

### Nasal symptoms evaluation and sample preparation

On Day 29, immediately after the final OVA provocation, nasal symptoms involving frequency of sneezes and nasal rubbing were assessed for 10 minutes. The mice were observed and reviewed independently by three blinded reviewers whose inter-interviewer variability was not statistically significant. After 24 hours, the mice were killed and the blood samples were harvested by cardiac puncture and were centrifuged to obtain serum for enzyme-linked immunosorbent assay (ELISA). Tracheas of the mice were partially resected and a 22-gauge catheter was inserted via the tracheal opening to the nasopharynx in order to perfuse the nasal passages from choana to nostril with 1 ml PBS. Nasal lavage fluid (NLF) was collected from the nares and centrifuged, and the supernatants were collected and stored at −80 °C for ELISA. Nasal mucosas were assayed by Periodic Acid-Schiff (PAS) stainning, toluidine blue staining, immunofluorescence staining, real-time PCR or western blotting methods.

### ELISA assay

Serum OVA-specific immunoglobulin E (IgE), histamine and leukotrienes C4 (LTC4) levels were assayed using specific ELISA kit (BlueGene Biotech, Shanghai, China) and concentrations of IL-4, IL-9 and IL-17 in NLF or IL-6 and IL-8 in the cell culture supernatants were assayed with specific ELISA kits (eBioscience Inc., USA). Each sample was analyzed in triplicate.

### PAS, toluidine blue and immunofluorescence staining

The mice were decapitated and the heads were fixed with 4% paraformaldehyde for 2 days and decalcified for 21 days in 10% ethylene diamine tetra acetic acid (EDTA).Then the samples were paraffin-embedded and serially sectioned. The sections were stained with PAS for counting goblet cell numbers, stained with toluidine blue for assessing mast cell infiltration, and stained with immunofluorescence for assessing KCa3.1 immunolabelling. Following deparaffinization and rehydration, the sections were rinsed using PBS for three times and then microwaved for antigen retrieval in EDTA buffer (PH8.0). The sections were incubated with primary antibody (rabbit anti-mouse KCa3.1, 1:100 dilution, Abcam) at 37 °C for 60 min, rinsed for three times and incubated with secondary antibody (cy3-labeled goat anti-rabbit IgG, 1:300 dilution, Abcam) for 30 min. Then, 4,6-diamidino-2-phenylindole (DAPI) was added for nuclear staining. Goblet cell, mast cell and KCa3.1 positive cell numbers were microscopically counted under high-powered field (HPF, ×400), and five fields of each section were randomly selected and mean value per HPF was calculated. All pathology slides images were reviewed independently by three blinded reviewers whose inter-interviewer variability was not statistically significant.

### RNA extraction and reverse transcription

After homogenization of the tissues or cells, TRIzol reagent was added to extract total RNA. The purity and integrity were assessed by measuring absorbance ratios at 260/280 nm (1.8 ~ 2.0 was considered eligible) and agarose gel electrophoresis, respectively. For reverse transcription, PrimeScript™ 1st Strand cDNA Synthesis Kit (TaKaRa) was applied to reversely transcribe two micrograms total RNA into cDNA as a template for real-time PCR.

### Real-time PCR

SYBR Green PCR master mix (TaKaRa) was employed to assay KCa3.1, mucin 5AC (MUC5AC) and tryptase mRNA levels. Primers were designed and synthesized (Sangon Biotech, Shanghai, China). Each sample was analysed in triplicate. KCa3.1 (120 bp) primers were: forward primer, 5–AAGCACACTCGA AGGAAGGA–3 and reverse primer, 5–ATTCACTTGTTCCCGGAGCT –3. MUC5AC (193 bp) primers were: forward primer, 5–AGGGCTCTGTGACAAC TACC–3 and reverse primer, 5–TGGGGTGTGGGTAGAAGAAC–3. Tryptase (142 bp) primers were: forward primer, 5–CCGCCACCATTTCCTTTGAA–3 and reverse primer, 5–GTCCTTCATTCCCAGCACAC–3.The internal control GAPDH (186 bp) primers were: forward primer, 5–CAACTCCCACTCTTCCACCT–3 and reverse primer, 5–CTTGCTCAGTGTCCTTGCTG–3. Relative mRNA level was expressed as the relative fold change and calculated using the formula: 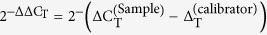 where each 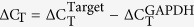. One sample from a normal control mouse was designated as a calibrator.

### Mast cell culture and intervention

Culture of the mouse mastocytoma cell line P815 cells (ATCC, Manassas, VA, USA) were performed as described previously^45^. Briefly, P815 cells were cultured with Dulbecco’s modified Eagle’s medium (DMEM, ATCC, Manassas, VA, USA) containing 10% fetal bovine serum (FBS), 1.5 mg/ml sodium bicarbonate, 4.5 mg/ml glucose, 4 mM L-glutamine, 100 U/ml penicillin and 100 μg/ml streptomycin at 37 °C in 5% CO2, humidified atmosphere. Before intervention, P815 cells were cultured in the serum-free basal medium for at least 24 h. The cells were divided into four groups and then incubated with 10 μl PBS (blank control), 10 μl negative-shRNA (1 × 10^8^ TU/ml, negative control), 10 μl 1 × 10^8^ TU/ml lentiviral KCa3.1-shRNA (Ksh), or 10 μl 1 × 10^8^ TU/ml lentiviral KCa 3.1-shRNA supplemented with 20 μM phosphatidylinositol 3-kinase (PI3K)/AKT signaling pathway inhibitor LY294002 (Cell Signaling Technology) for 12 h, respectively. Then, the cell pellets were harvested for assaying KCa3.1, p-AKT and AKT protein levels using western blotting and tryptase mRNA levels using real-time PCR and the culture supernatants were centrifuged and collected for detecting IL-6 and IL-8 protein levels by ELISA.

### Western blotting

The cells were homogenized and lysed in RIPA buffer (Pierce) and then centrifugated at 14000 rpm for 15 min at 4 °C. The supernatant involving the total protein were collected. Following quantification of protein concentration, the samples were loaded on SDS–PAGE gels (Invitrogen) for protein separation and subsequently electroblotted onto PVDF membranes (Millipore) under the same experimental conditions. After blocking of excess protein-binding sites with 5% skimmed milk for 1 h, the PVDF membranes were incubated with the primary antibody (rabbit anti-mouse KCa3.1, 1:1000 dilution, Abcam; rabbit anti-mouse p-AKT and AKT, 1:1000 dilution, Cell Signaling Technology) at 4 °C overnight. After TBST washing, the secondary goat anti-rabbit IgG-HRP antibody (Abcam) was added for 1 h incubation. Lastly, immunoreactions were visualised by the ECL kit (Pierce). β-actin was used as an internal control. Densitometric analyses were performed using Gel-Pro Analyzer 4.0 software (Media Cybernetics).

### Statistical analysis

Statistical analysis softwares (GraphPad Prism 5.0 and SPSS version 20.0 software) were applied to conduct the analyses. One-way ANOVA test followed by Dunnett’s tests were employed for intergroup comparison of all the continuous variables for them had normal distribution. Correlations were assessed by Spearman’s test. Values were shown as mean ± standard error (SE). Two-sided P value < 0.05 was deemed statistically significant.

## Additional Information

**How to cite this article**: Lin, H. *et al.* Lentiviral shRNA against KCa3.1 inhibits allergic response in allergic rhinitis and suppresses mast cell activity via PI3K/AKT signaling pathway. *Sci. Rep.* **5**, 13127; doi: 10.1038/srep13127 (2015).

## Supplementary Material

Supplementary Information
