# Supplementary material for: Lentiviral shRNA against KCa3.1 inhibits allergic response in allergic rhinitis and suppresses mast cell activity via PI3K/AKT signaling pathway
Source: Sci Rep. 2015 Aug 14;5:13127. doi: 10.1038/srep13127 (PMC4536635; doi:10.1038/srep13127)
Supplement: Supplementary Information [file srep13127-s1.doc]

**Lentiviral shRNA against KCa3.1 inhibits allergic response in allergic rhinitis and suppresses mast cell activity via PI3K/AKT signaling pathway**

**Hai Lin ^1,2^, Chunquan Zheng ^2,*^, Jing Li ^2,3^, Chen Yang ^2,4^ & Li Hu ^5^**


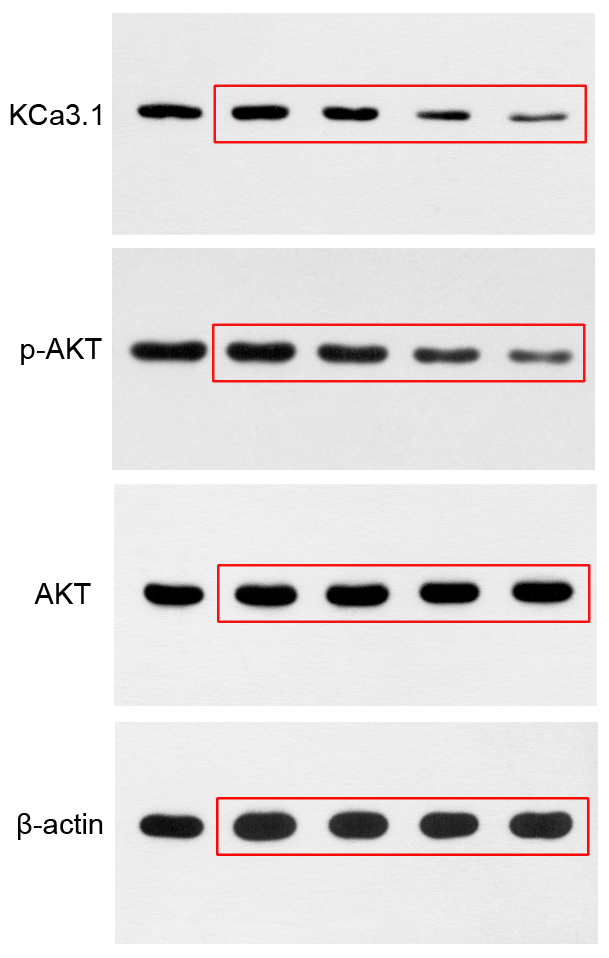


Supplementary figure legend

**Supplementary Figure 1.** Full-length blots images for western blotting results.
